# Twists and turns: spontaneous torsion of accessory liver lobe and gallbladder—diagnostic challenges and surgical interventions—a case report

**DOI:** 10.3389/fped.2025.1530918

**Published:** 2025-03-06

**Authors:** Soumaya Charaa, Julien Frandon, Ghizlane Touimi Benjelloun, Benoit Tessier

**Affiliations:** ^1^ Department of Radiology and Medical Imaging, Nîmes University Hospital Center, University of Montpellier-Nîmes, Nîmes, France; ^2^ Medical Imaging Group Nîmes (MIG Nîmes), Nîmes, France; ^3^ Department of Pediatric Surgery, Lapeyronie Hospital, Montpellier University Hospital Centre, University of Montpellier, Montpellier, France; ^4^ Institute Desbrest of Epidemiology and Public Health, INSERM UMR 1302, University of Montpellier, Montpellier, France

**Keywords:** accessory liver lobe, torsion, liver, surgery, computed tomography, ultrasound, case report

## Abstract

An accessory liver lobe (ALL) represents a rare congenital variation of liver tissue, typically resulting from focal excessive development of liver tissue. Torsion of such lobes, though rare, can precipitate a severe surgical crisis due to hepatic ischemia and failure. We report a case involving a 9-year-old patient who was admitted with acute epigastric pain. Doppler ultrasound and contrast-enhanced CT scans revealed a heterogeneous, avascular mass with displacement of the gallbladder, which had a thickened wall. During laparotomy, a twisted, congested ALL along with the gallbladder was surgically removed. Diagnostic imaging, particularly computed tomography (CT), plays a crucial role in the rapid identification of causes behind acute abdominal pain, necessitating meticulous analysis of CT scans. We share the findings from imaging and surgery to enhance awareness of this rare condition.

## 1 Introduction

An accessory liver lobe (ALL) is a rare congenital anomaly that occurs in <1% of the population (1, 2). ALL is formed by a healthy liver parenchyma that has anatomical continuity with the normal liver. Riedel's lobe is the most commonly recognized form of ALL (1). Ridel’s lobe is a tongue-like elongation of hepatic segments V and VI (1). The origin is a congenital dysembrioplastic anomaly in the development of a hepatic bud, which can lead to the formation of accessory lobes (3). Patients with ALL are generally asymptomatic. However, ALL can result in liver torsion, requiring surgical resection, due to the risk of hepatic ischemia and hepatic failure (2, 4). Computed tomography (CT) is crucial for prompt diagnosis, and CT images should be carefully examined. Despite its frequent appearance in animals, such as dogs and rabbits, this complication is rarely observed in humans (5).

ALL are also associated with persistent defects of the anterior abdominal wall including omphalocele, Beckwith–Wiedemann syndrome, umbilical hernia, and cloacal exstrophy. To our knowledge, there has never been a case of torsion of a hepatic accessory lobe in a child with a history of right diaphragmatic hernia.

This case report emphasizes the importance of using the right imaging approach to diagnose and treat accessory hepatic lobe torsion.

This manuscript was prepared following the CARE guidelines (https://www.care-statement.org).

## 2 Case report

A 9-year-old boy was admitted to the emergency department with complaints of epigastric pain and emesis, which suddenly emerged and exacerbated over the last few hours. He had an emergency right diaphragmatic hernia repair during the neonatal period. During this first surgery, the accessory liver segment was not described. At the examination, he was found to be pale and had a tachycardic rate of 100 bpm. He was afebrile. There was a diffuse tenderness in the abdomen, mostly in the right upper quadrant. Gentle palpation revealed an abdominal mass in the right upper quadrant. Blood tests demonstrated marked elevation of liver function [ALT: 404 U/L (normal values: <40 U/L), AST: 500 U/L (normal values: <45 U/L), gamma-glutamyltransferase (GGT): <70 U/L (normal values: 0 ± 30 U/L), and ALP: 350 U/L (normal values: <140 U/L)]. C-reactive protein level was normal.

**Figure 1:**
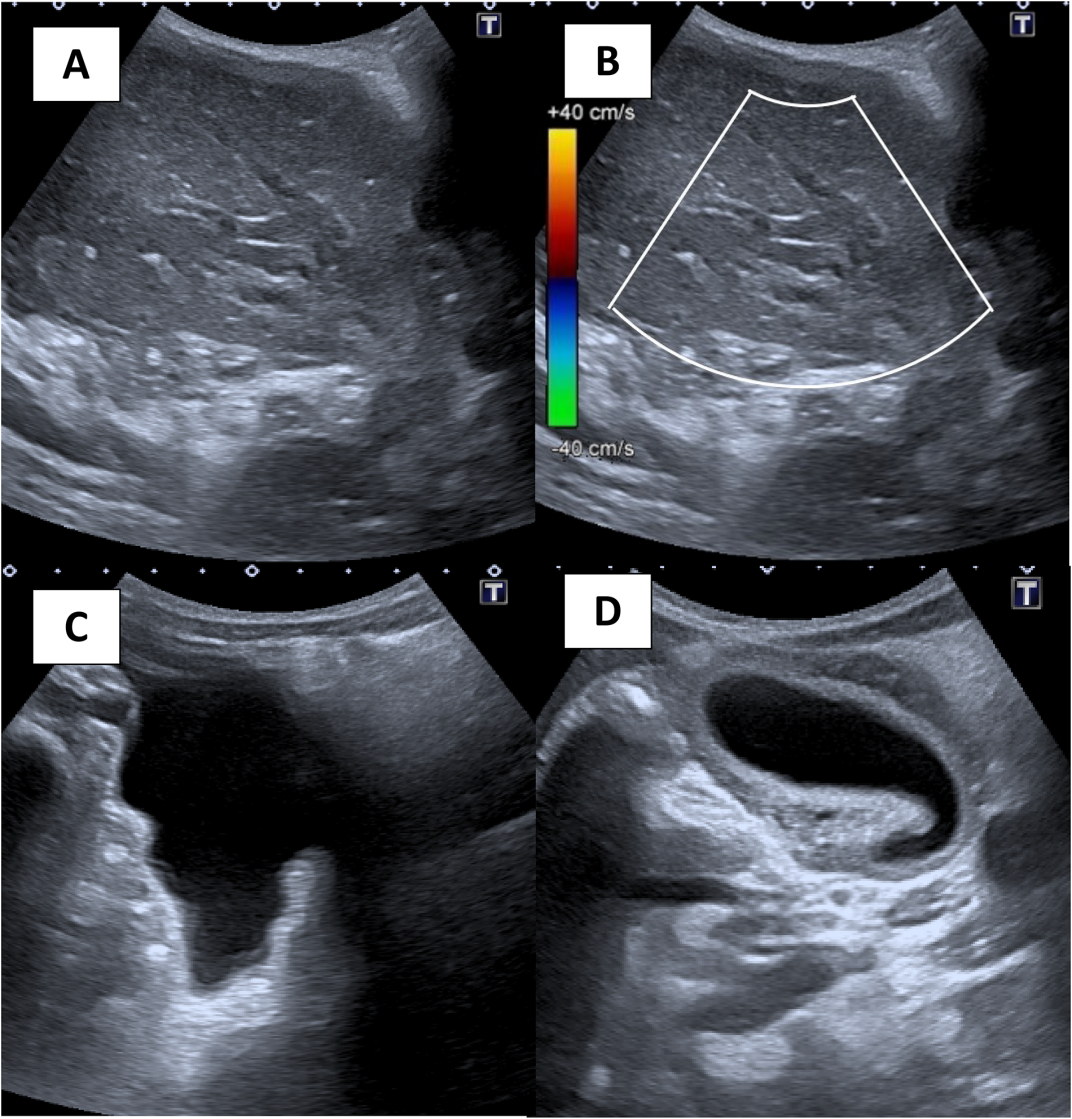
**(A)** grayscale ultrasound image of the right abdomen shows a mass that is mostly hypoechoic and heterogeneous. **(B)** Color flow Doppler image demonstrates no color flow within the mass. **(C)** Free fluid in the pelvis. **(D)** A gallbladder that is displaced to the left and with a thick wall.

Ultrasound (US) revealed a large midabdominal mass of mixed echogenicity, with no flow on color Doppler interrogation, displacing the retroperitoneal vessels and the pancreas posteriorly. A moderate amount of fluid was found in the left upper quadrant and pelvis. The gallbladder was displaced to the right and had a thick wall (Figures 1A–D).

**Figure 2:**
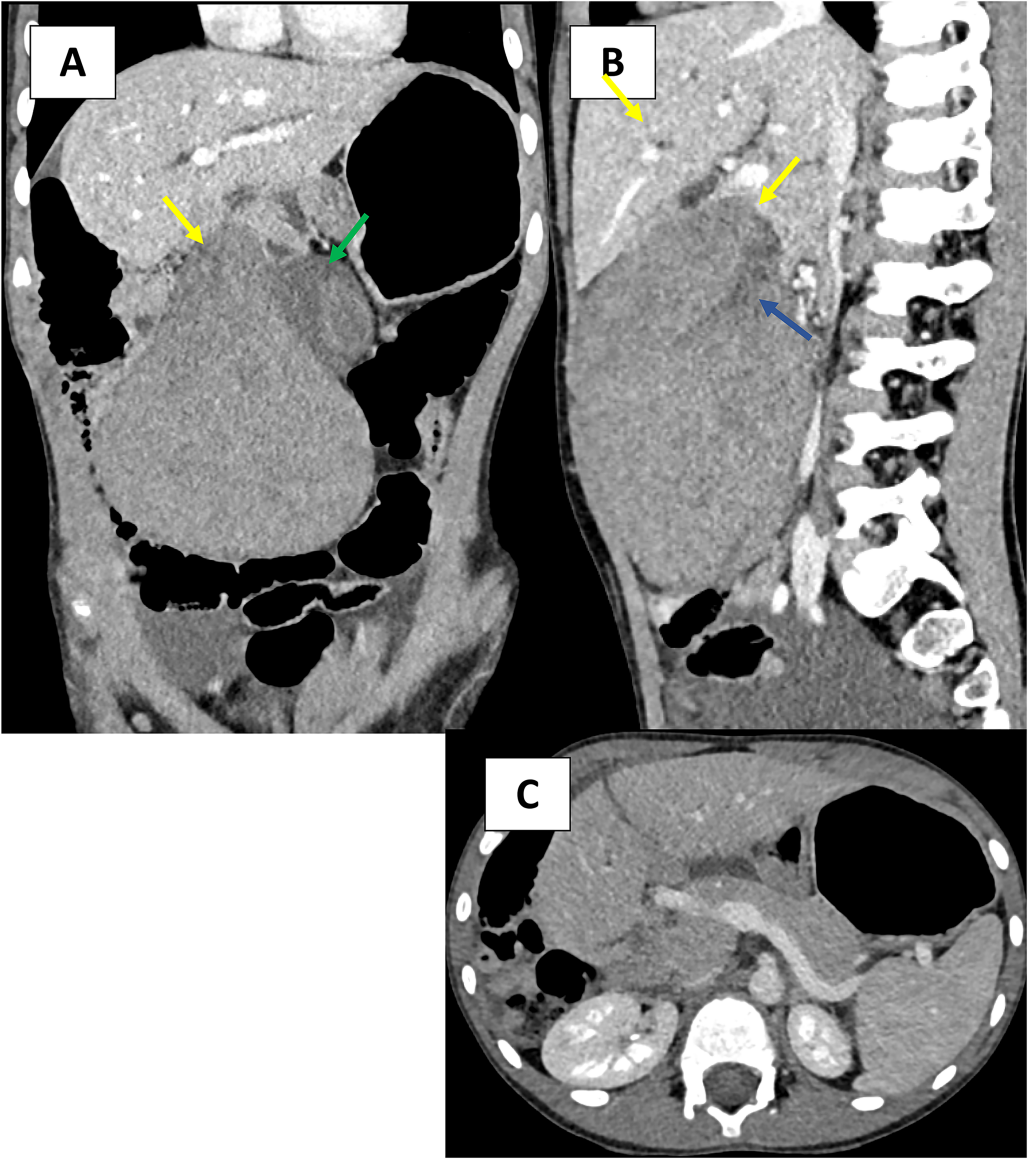
**(A–C)** Contrast-enhanced, axial, coronal, and sagittal CT images in the venous phase demonstrate a large, heterogenous low-attenuation mass in the right abdomen. **(A–C)** The mass shows tubular structures, likely a thrombosed portal vein (blue arrow) extending into the thin pedicle of the torsed ALL (yellow arrow). The gallbladder is displaced to the left and with a thick wall (green arrow).

CT without and with contrast demonstrated a heterogenous mass in the right upper quadrant. There was no enhancement following contrast administration. The gallbladder was displaced to the left, had a thick wall, and was surrounded by fluid. There was no evidence of free air. We suspected a Whirlpool sign on the underside of the liver (Figure 2).

After further discussion between the radiology and surgical teams, the patient was taken emergently to the operative room for exploratory laparotomy.

**Figure 3:**
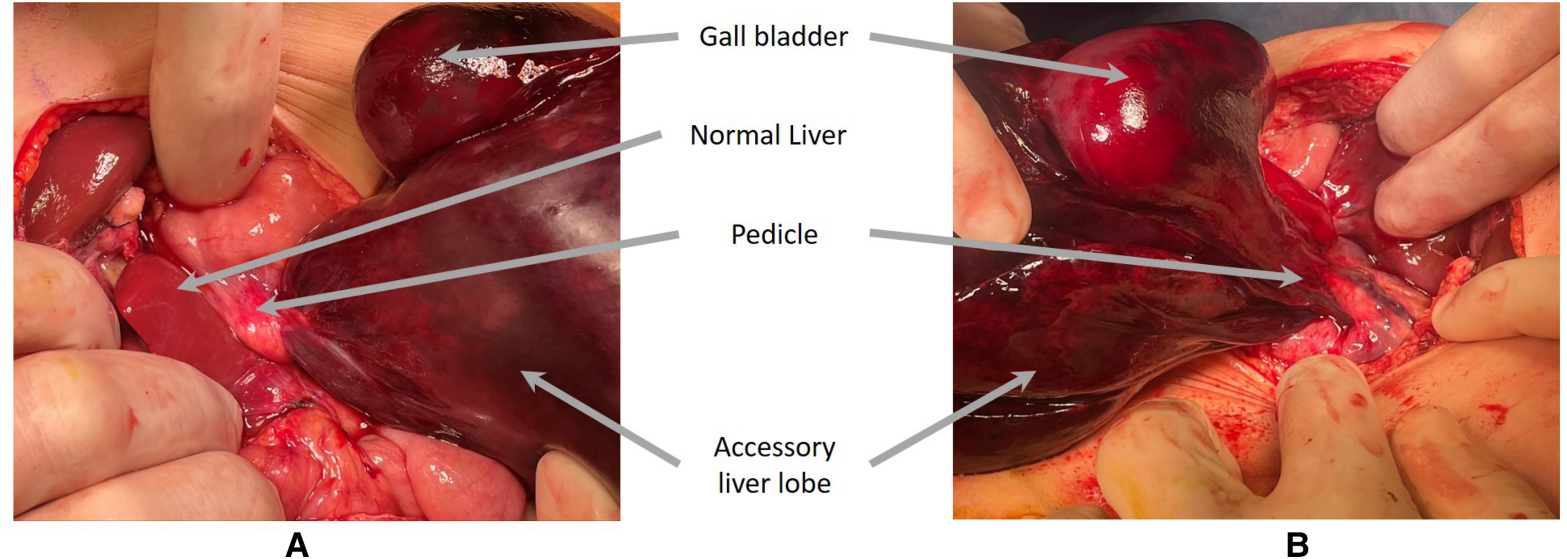
Intraoperative image of the twisted lobe and gallbladder before **(A)** and after **(B)** detorsion.

**Figure 4:**
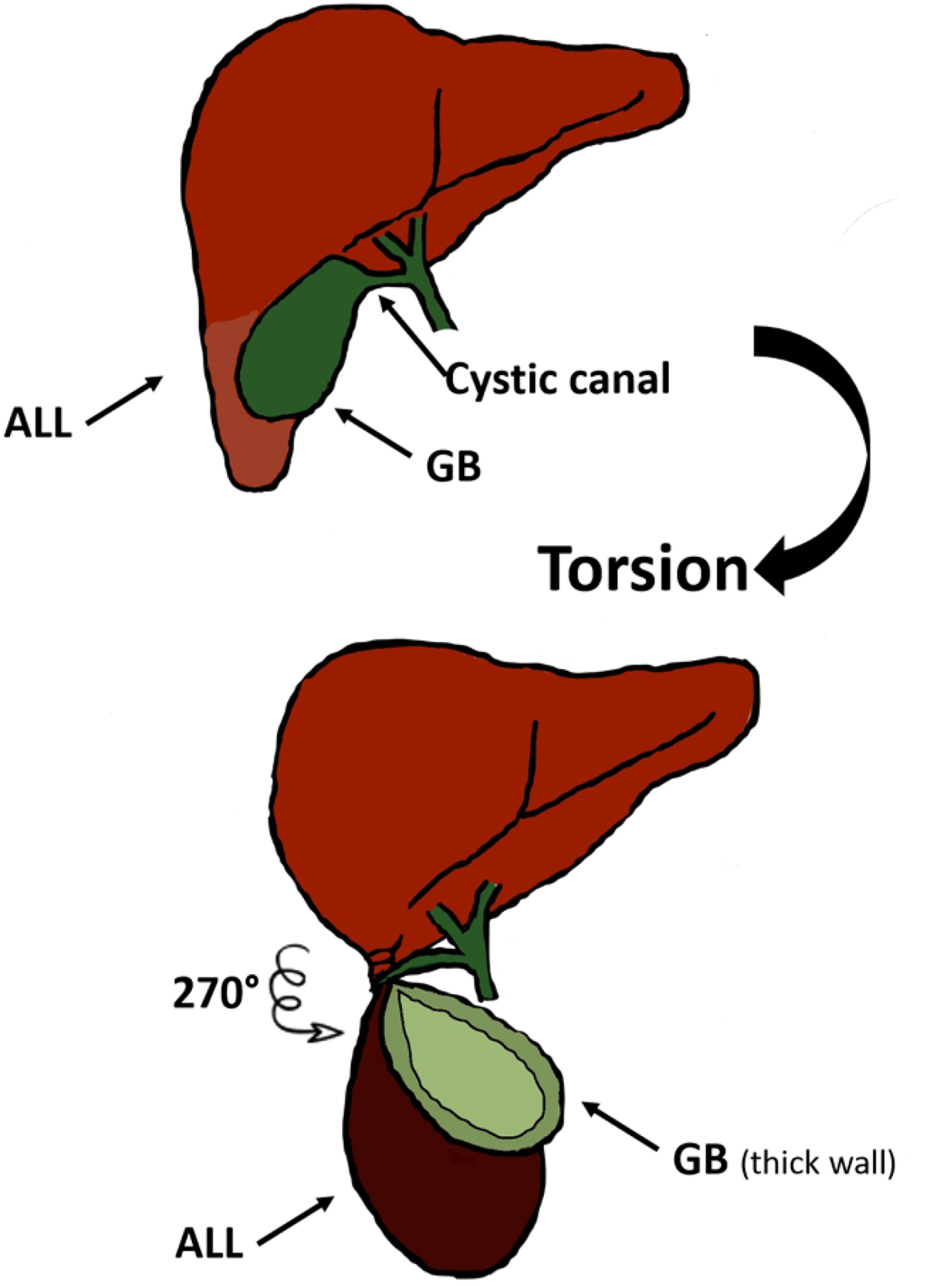
Image showing the mechanism of torsion of the ALL and cystic duct, explaining the gallbladder ischemia along with the accessory lobe.

The surgery was performed by a pediatric surgical team in association with an adult surgical team specializing in biliary surgery. A right supraumbilical transverse laparotomy confirmed the presence of an accessory liver segment with a twist. The gallbladder was dependent on the accessory hepatic lobe (segments V and VI). They were both ischemic. The lobe was twisted around a pedicle of artery, vein, and duct (Figures 3, 4). The pedicle was detorsed 270°.

**Figure 5:**
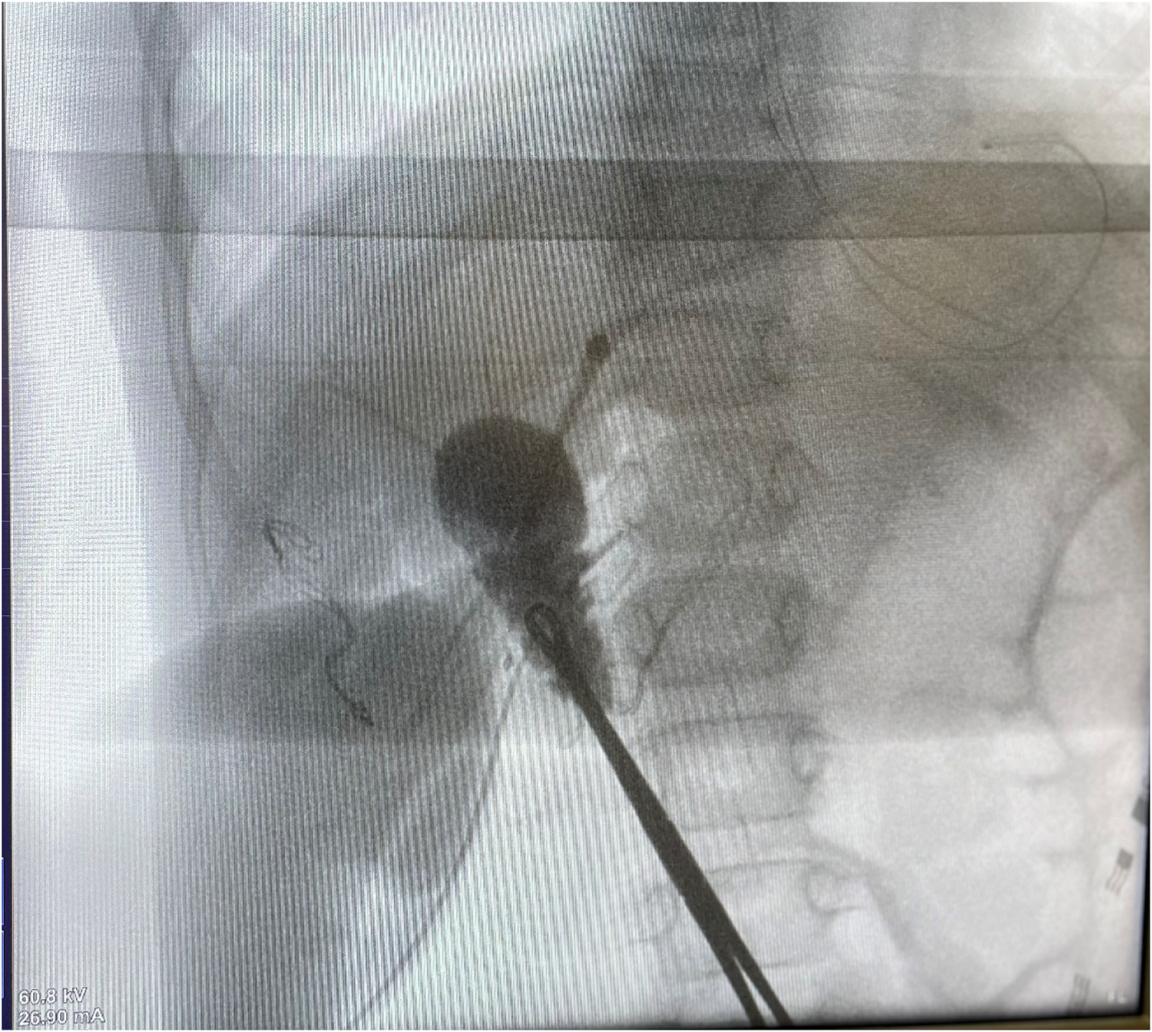
Cholangiography shows the integrity of the choledochal.

Cholangiography was performed prior to resection to check the integrity of the choledochal (Figure 5). The mass was excised by ligation of the pedicle.

Cholecystectomy was performed at the same time. The histology revealed normal mature liver tissue and no tumoral or specific lesion on the gallbladder.

The patient was discharged home on Postoperative Day 6 with a normal liver function test and normal US.

He was admitted to the hospital again for biloma and dilatation of the bile ducts 10 days after the surgery. US and CT scans were done to confirm the diagnosis. The blood test showed a sub-normal rise in liver enzymes [ASAT (52), ALAT(49), GGT(108)]. It can be explained by the fact that during the ligation of the bile ducts for the accessory lobe, a bile leak may have formed. Moreover, the ligation may also have caused a stenosis leading to a slight dilatation of the bile ducts. No treatment was given, with spontaneous regression of the collection and dilatation. The prognosis was satisfactory at the postoperative 6-month follow-up.

## 3 Discussion

The presence of accessory hepatic lobes is a rare congenital malformation with only a few case reports in the literature that describe their presentation (2). In some reports, their incidence is <1% of the population, but that number may be underestimated since most people are asymptomatic without any clinical consequence. They are identified incidentally on imaging or during other surgical procedures, where they are often mistaken for tumors (6). A close follow-up would be advisable in asymptomatic cases without surgery.

Most ALLs are located in the abdominal cavity but can be found in the thoracic cavity (7). Accessory liver lobes are defined as supernumerary liver lobes, composed of normal liver parenchyma in continuity with the liver, in contrast to ectopic liver lobes that have no anatomical continuity with the normal liver (1, 5). They can be sessile or pedunculated depending on their type of attachment to the liver (1).

The Riedel’s lobe, an elongated inferior right liver lobe, is the most common and well-known place for an ALL (8).

Despite its frequent appearance in animals, such as dogs and rabbits, this complication is rarely observed in humans, and the preoperative diagnosis can be challenging (4–6).

The liver has multiple ligamentous connections to the abdominal wall and diaphragm (5). This includes the coronary ligament, triangular ligament, and the falciform ligament. In the few cases of ALL torsion that have been reported, several cases have occurred in patients who have a history of omphalocele repair (5, 9). A hepatic torsion could be caused by the lack of a ligamentous attachment, either surgical or congenital, and a history of omphalocele repair should be considered (5). In the case of a right diaphragmatic hernia, there is no attachment between the liver and the diaphragm by ligament which could facilitate a torsion. To our knowledge, this is the only case in the literature that describes ALL torsion with a history of right diaphragmatic hernia repair. In the case of a diaphragmatic hernia, a search for the accessory lobe could be carried out. Even if this would have no surgical consequences, it would provide information to parents and improve management in the event of complications.

Most patients with a twist are presented with right-side abdominal pain accompanied by nausea or vomiting. Blood tests show abnormal liver function. Ultrasonography and CT are crucial for prompt diagnosis, and CT images should be carefully examined.

The US images of our patient showed a heterogeneously hypoechoic mass with no flow on color, and echogenic linear structures suspected to be thrombosed portal veins.

The CT images demonstrated a heterogeneous, non-enhanced mass, extending from hepatic segments V and VI. On coronal images, we described the presence of non-enhancing vascular structures extending into a thin pedicle with a whirlpool sign. The gallbladder was displaced to the left and had a thick wall.

US and CT can be confusing due to the loss of internal architecture of the mass caused by infarction. When congested, the mass may appear hypoechoic compared to normal liver tissue due to the increase in fluid content and the loss of its vascular supply. There was no flow within the mass during color Doppler US or after intravenous contrast in the CT examination (5).

Prompt management and emergency excision are recommended in case of suspected ALL torsion due to the risk of hepatic ischemia and hepatic failure (2, 4).

Lowther et al. found that all cases (37 cases), including ours, required hepatectomy, and thus, the surgical teams need to be prepared to perform these complex resections (2).

The existence of primary hepatocellular or metastatic tumors in accessory or ectopic lobes necessitates a careful study of CT images (1).

## 4 Conclusion

It’s crucial for the radiologist to recognize this condition as a cause of sudden abdominal pain in the right upper quadrant. The overall morbidity can be reduced by preoperative diagnosis of this abnormality and mapping its vascular and biliary anatomy.

## Data Availability

The original contributions presented in the study are included in the article/Supplementary Material, further inquiries can be directed to the corresponding author.
